# Objective Assessment of Cognition for Detecting Subjective Cognitive Decline Is More Accurate than Subjective Estimations: The Role of Trait Affect

**DOI:** 10.3390/jintelligence13090118

**Published:** 2025-09-11

**Authors:** Nikoleta Frantzi, Despina Moraitou, Eudokia Emmanouilidou, Eleni Poptsi, Emmanouil Tsardoulias, Andreas L. Symeonidis, Georgia Papantoniou, Maria Sofologi, Elvira Masoura, Glykeria Tsentidou, Ioanna-Giannoula Katsouri, Magda Tsolaki

**Affiliations:** 1 Laboratory of Psychology, Department of Cognition, Brain and Behavior, School of Psychology, Aristotle University of Thessaloniki (AUTh), 54124 Thessaloniki, Greece; despinamorait@gmail.com (D.M.); eudokiaemman@gmail.com (E.E.); emasoura@psy.auth.gr (E.M.); gltsentidou@gmail.com (G.T.); 2 Laboratory of Neurodegenerative Diseases, Center for Interdisciplinary Research and Innovation, Aristotle University of Thessaloniki (CIRI—AUTh), 54124 Thessaloniki, Greece; poptsielena@gmail.com (E.P.); tsolakim1@gmail.com (M.T.); 3 Greek Association of Alzheimer’s Disease and Related Disorders (GAADRD), Petrou Sindika 13 Str., 54643 Thessaloniki, Greece; 4 School of Electrical and Computer Engineering, Faculty of Engineering, Aristotle University of Thessaloniki (AUTh), 54124 Thessaloniki, Greece; etsardou@ece.auth.gr (E.T.); asymeon@eng.auth.gr (A.L.S.); 5 Laboratory of Psychology, Department of Early Childhood Education, School of Education, University of Ioannina, 45110 Ioannina, Greece; gpapanto@uoi.gr (G.P.); m.sofologi@uoi.gr (M.S.); 6 Department of Occupational Therapy, Faculty of Health and Caring Sciences, University of West Attica, 12243 Athens, Greece; ykatsouri@uniwa.gr

**Keywords:** Alzheimer’s disease (AD), executive functions, memory estimation, negative affect, personality positive affect

## Abstract

The early identification of cognitive decline is crucial for well-timed intervention and diagnosis, particularly in the context of preclinical Alzheimer’s disease (AD). In this study, we investigated the complex interplay between trait affect, objective cognitive performance, and subjective memory estimations in a sample of 105 older adults. Using path analysis, we aimed to determine whether trait affect and objective cognitive control abilities predict individuals’ subjective perceptions of their own memory abilities. The results revealed that both positive and negative trait affect significantly predicted subjective memory estimations, while objective cognitive control performance did not significantly predict these estimations. These findings highlight a crucial dissociation between objective and subjective cognitive measures. Therefore, the present results underscore the critical importance of complementing self-reported cognitive estimations, which can be biased by stable emotional dispositions, with objective cognitive tools like the R4Alz-pc (preclinical) index. This approach enables a more accurate evaluation of cognitive health in advancing age, especially for the early detection of subtle dysfunction in preclinical AD.

## 1. Introduction

With population aging, identifying Alzheimer’s disease (AD) in its earliest, pre-dementia stages has become a major focus of research. Increasing attention has been directed toward the preclinical stage of AD, which includes both Mild Cognitive Impairment (MCI) and Subjective Cognitive Decline (SCD), characterized by subtle cognitive changes that often precede clinically measurable impairment (Jessen et al. 2014). Importantly, these cognitive alterations may co-occur with underlying biological changes. Amyloid-β deposition, tau pathology, and neurodegenerative processes have all been observed to begin years before clinical diagnosis, supporting the view of AD as a long, progressive disease continuum (Dubois et al. 2023; Jack et al. 2018; Sperling et al. 2011; Verfaillie et al. 2019). Neuroimaging and cerebrospinal fluid (CSF) studies have consistently demonstrated such biomarker abnormalities in asymptomatic individuals with SCD or MCI, suggesting that pathological processes are already active during this preclinical window (Dubois et al. 2016; Jessen et al. 2014). Recent models have conceptualized preclinical AD not as a discrete phase, but rather as a dynamic continuum with variable trajectories and heterogeneous clinical manifestations (Jack et al. 2018), emphasizing the need for reliable cognitive and behavioral indicators, capable of capturing these preclinical manifestations.

Specifically, AD is now understood as a progressive condition that unfolds over many years, with MCI often representing its first clinically detectable phase (Scheltens et al. 2021). MCI is characterized by measurable cognitive decline without significant functional impairment (Petersen et al. 2014). It is typically classified into amnestic and non-amnestic subtypes, depending on whether memory or other cognitive domains are primarily affected. Furthermore, MCI can be single-domain or multiple-domain, with the latter often predicting a more rapid progression to dementia (Albert et al. 2011). Among the cognitive deficits observed in MCI, episodic memory and executive functioning appear particularly vulnerable. In fact, impairments in executive processes are especially prominent in individuals with multi-domain amnestic MCI and are considered reliable indicators of progression toward AD (Johns et al. 2012; Ready et al. 2003).

Even earlier in this continuum lies SCD. SCD, often described in the literature as subjective memory complaints, refers to a self-perceived deterioration in memory and/or other cognitive abilities compared to a previous level of functioning, despite intact performance on “traditional” standardized neuropsychological tests (Jessen et al. 2014, 2020; Rabin et al. 2017). Although such self-perceptions do not always indicate underlying neurodegenerative processes, SCD, when persistent and accompanied by biomarker evidence or specific risk factors, may reflect the initial clinical expression of preclinical AD (Jessen et al. 2020; Si et al. 2020). Research indicates that SCD can manifest for several years, even decades, before the clinical onset of dementia, highlighting a critical window for early intervention and disease modification strategies (Drabo et al. 2025; Jessen et al. 2020). Nevertheless, SCD is a heterogeneous construct, and its interpretation remains debated, as psychological factors and personality traits may also contribute to subjective complaints, especially in the absence of neuropathology (Hill et al. 2016; Jenkins et al. 2021; Schlosser et al. 2020). However, it should be considered that according to the diagnostic criteria proposed by the SCD-I Working Group, feelings of worsening memory performance should not be associated with the presence of depressive symptoms (Jessen et al. 2014). Accordingly, multidimensional approaches to SCD assessment, integrating cognitive, stable psychological traits, and biological data, are essential to disentangle its complex etiology.

Findings on the relationship between SCD and objective cognitive performance have been inconsistent (Burmester et al. 2016). This inconsistency may stem from the heterogeneity of SCD, methodological differences across studies, or the lack of sufficiently sensitive neuropsychological tools to detect subtle cognitive changes in preclinical AD (Jessen et al. 2014; Kuhn et al. 2021; Poptsi et al. 2019, 2024). Although SCD is defined by the absence of measurable cognitive deficits, several studies have reported associations between self-reported cognitive complaints and objective performance, particularly in domains of executive functioning (Harrington et al. 2013; Poptsi et al. 2024; Rao et al. 2025; Webster-Cordero and Giménez-Llort 2022). Executive functions are higher-order cognitive abilities essential for coordinating various cognitive and emotional processes and adapting to novel or challenging situations. Several influential theoretical models have been proposed to explain the complex nature of executive functions. Miyake et al. (2000) introduced the “unity and diversity” model, which conceptualizes executive functions as partially distinct but heavily interrelated abilities, including task switching, working memory updating, and inhibitory control. Complementarily, Diamond (2013) emphasized the same core components, that is, working memory, inhibition, and cognitive flexibility, as foundational to executive functioning. Lavie’s load theory (Lavie et al. 2004) focuses on how perceptual and cognitive load influence selective attention, suggesting that attentional control mechanisms adapt depending on task demands to suppress irrelevant information. Engle and Kane (2003) proposed a two-factor model linking working memory capacity with attentional control, particularly under conditions of distraction. Cooperatively, these frameworks underscore the multifaceted nature of executive functions and their critical involvement in goal-directed behavior and cognitive regulation.

Executive functions are often among the earliest cognitive domains to show vulnerability in aging, even before memory impairment (Huff et al. 2015; Mulligan et al. 2016; Pantsiou et al. 2018; Tsentidou et al. 2022), and are closely linked to preclinical AD, including MCI or even SCD (Harrington et al. 2013; Huff et al. 2015; Webster-Cordero and Giménez-Llort 2022). Recent studies have consistently reported subtle, yet significant, impairments in various aspects of executive functions in individuals within the preclinical AD spectrum, when compared to cognitively healthy older adults. More specifically, a recent meta-analysis by Rabi et al. (2020) revealed a common generalized inhibition deficit among people with amnestic MCI (aMCI). Correspondingly, individuals with SCD exhibit poorer performance in executive functions compared to control groups, particularly in inhibition (López-Higes et al. 2025; Pérez-Cordón et al. 2020; Poptsi et al. 2020, 2023, 2024; Rao et al. 2025), and in task-switching ability (Poptsi et al. 2020, 2023, 2024; Wolfsgruber et al. 2020). Broader executive deficits have also been reported, including attention, working memory, cognitive flexibility, planning, monitoring, verbal fluency, and decision-making (Webster-Cordero and Giménez-Llort 2022). Recent work by Poptsi et al. (2023) shows that individuals with SCD performed significantly lower on cognitively demanding executive tasks, such as task switching and cognitive flexibility, suggesting that such deficits may be observable even in the absence of measurable global decline.

These findings are supported by neuroimaging evidence, indicating that SCD is associated with alterations in brain regions critical for executive functioning, particularly the frontal lobe (Mattsson et al. 2014) and prefrontal cortex (Vlassenko et al. 2011). These areas are among the earliest to exhibit functional and structural disruptions among individuals at risk for AD (Valech et al. 2017). A recent systematic review found a direct relationship between subjective cognitive complaints and atrophy of the orbital prefrontal regions, reduced functional connectivity in the retrosplenial–precuneus regions, and reduced temporal cortical thickness in the bilateral hippocampi and left frontal regions, all associated with decreased executive performance (Webster-Cordero and Giménez-Llort 2022. Given that executive dysfunction is a key marker of early neurodegenerative processes, assessing these functions in individuals with SCD may provide clinically useful insights into who is at greater risk of progressing toward MCI or AD dementia (Jessen et al. 2014).

However, self-reported memory complaints often fail to align with objective test performance, with many studies finding weak or non-significant correlations between subjective reports and neuropsychological outcomes (Reid and MacLullich 2006). This discrepancy has prompted investigation into additional factors, such as metacognition and personality traits, which may shape the subjective experience of decline. Metacognition, or the capacity to monitor and evaluate one’s own cognitive functioning, underlies people’s judgments of their memory and thinking (Efklides 2011; Flavell 1979; Jenkins et al. 2021). When metacognitive monitoring is compromised, individuals may systematically over- or underreport cognitive lapses despite normal objective performance (Bampa et al. 2023). Importantly, stable personality traits may influence these metacognitive evaluations (Colvin et al. 2018). For instance, there is consistent evidence that neuroticism is associated with increased cognitive complaints and subjective estimations about cognitive failures. (Aschwanden et al. 2018; Könen and Karbach 2018; Lange and Süß 2014; Snitz et al. 2015). This relationship may be attributed to a tendency for individuals high in neuroticism to adopt a pessimistic filter, amplifying memory concerns (Sutin et al. 2020), whereas those with strong conscientiousness or extraversion are more likely to maintain accurate or optimistic self-views. Consequently, higher conscientiousness has been found to correlate with fewer cognitive complaints (Könen and Karbach 2018; Snitz et al. 2015). Indeed, individuals scoring high in extraversion, openness, or conscientiousness tend to report fewer cognitive complaints, perhaps because they interpret minor lapses as inconsequential, perceiving their memory more favorably, even when objective performance is relatively lower (Buratti et al. 2013; Hülür et al. 2015). Yet, limited research has directly examined how these enduring affective dispositions may influence the complex relationship between objective cognitive performance and subjective estimations of decline. Crucially, diminished awareness of objective cognitive decline risks delayed diagnosis and foregoes opportunities for early, impactful intervention (Costa et al. 2023).

Drawing from this theoretical framework and the identified research gap, despite the recognized importance of affective factors, the precise influence of stable affective tendencies on the relationship between subjective and objective cognitive status remains underexplored. Therefore, the present study aims to investigate this relationship by focusing on the role of trait affect.

Trait affect refers to stable, enduring patterns in how individuals experience emotions across time and different situations, representing a fundamental aspect of personality assessment (Watson et al. 1988). Unlike temporary emotional states, trait affect captures how people generally feel or their typical emotional experiences over extended periods. This construct is typically measured through two primary dimensions: Positive Affect (PA) and Negative Affect (NA). Trait PA mainly corresponds to the personality dimension of extraversion and reflects a person’s enduring tendency to experience enthusiasm, energy, and engagement, while trait NA primarily aligns with anxiety/neuroticism and represents an individual’s consistent predisposition to experience negative emotional states, such as distress and unpleasurable engagement (Watson and Walker 1996). These trait dimensions are relatively independent of each other and have been linked to fundamental differences in how people respond to environmental stimuli (Watson et al. 1988). Understanding the role of trait affect in this context is crucial. Unlike transient mood states, dispositional affect that reflects enduring personality traits may bias both information processing and self-evaluations.

### Aim and Hypotheses of the Study

The present study aimed to examine the relationship between subjective estimations of cognitive impairment and objective cognitive performance in adults of advancing age, with a particular focus on the role of trait affect as a potential mechanism that can affect both objective assessment and subjective estimation of cognition.

The hypotheses of the study were formulated as follows:

**Hypothesis** **1:** *Both negative and positive trait affect will significantly predict subjective estimations of cognitive functioning in older adults.*

**Hypothesis** **2:** *Both negative and positive trait affect will significantly predict objective performance on measures of cognitive functioning in older adults.*

## 2. Materials and Methods

### 2.1. Participants

Power analysis was initially conducted using G*Power3.1.9.7 (Faul et al. 2007) for the F-test: Linear multiple regression. The results suggested a sample size of at least 68 participants to achieve a power of 0.80, assuming two predictors and a medium effect size of 0.15. However, because the study included path analysis within a structural modeling context, a larger sample was considered necessary to ensure sufficient statistical power and model fit. Participants were recruited via snowball sampling, primarily through announcements posted on social media. The inclusion criteria were as follows: age over 45 years and absence of any self-reported neurological or psychiatric diagnosis. The final sample consisted of 105 community-dwelling Greek adults (70 women and 35 men), aged 45 to 74 years (M = 59.25, SD = 5.21 years; see Table 1).

All participants were native Greek speakers and shared the same cultural background. In terms of education, each participant completed at least 13 years of formal schooling, indicating a relatively high level of educational attainment. It is important to note that none of the individuals had previously undergone clinical evaluation for preclinical AD pathology. As such, the potential presence of undetected cases of MCI or SCD within the sample cannot be ruled out.

### 2.2. Instruments

#### 2.2.1. The Positive and Negative Affect Schedule (PANAS: Trait Version)

Trait affect was measured using the Greek version of the Positive and Negative Affect Schedule (PANAS; Watson et al. 1988), as adapted and validated by Moraitou and Efklides (2009). The PANAS is a self-report questionnaire consisting of 20 items, 10 assessing positive affect, such as enthusiasm, determination, and pride, and 10 assessing negative affect, such as fear, nervousness, and hostility. Participants were asked to indicate to what extent they generally tend to feel each emotion on a Likert-type scale from 1 (very few times or not at all) to 5 (too many times). The Greek version confirmed the original two-factor structure comprising positive affect and negative affect as discrete, broader dimensions of emotionality. The internal consistency of the two scales was satisfactory, with Cronbach’s alpha values of 0.84 for positive affect and 0.82 for negative affect.

#### 2.2.2. Multifactorial Memory Questionnaire (MMQ): Ability Scale

SCD was assessed using the MMQ-Ability scale, one of the three subscales of the Multifactorial Memory Questionnaire (MMQ; Troyer and Rich 2002), in its Greek version (Emmanouilidou et al. 2023). This subscale assesses perceived memory ability in everyday life. MMQ is a self-report questionnaire consisting of 57 items, designed for use by clinicians and researchers to assess metamemory in middle-aged and older adults. It comprises three subscales: overall contentment or satisfaction with one’s own memory ability (MMQ-Contentment), perceived memory ability (MMQ-Ability), and use of everyday memory strategies and aids (MMQ-Strategy).

In the present study, only the MMQ-Ability subscale was used. It contains 20 items referring to everyday memory situations, presented as examples of memory failures (e.g., “How often do you forget an appointment?” or “How often do you misplace something you use daily, like your keys or glasses?”). Participants rated how often each situation applied to them over the past two weeks, using a five-point Likert scale ranging from 0 = All the time to 4 = Never. A total score was calculated by summing all responses. Higher scores indicate better subjective memory ability.

To examine the structural validity and internal consistency of the Greek version of the MMQ-Ability scale, exploratory factor analysis (EFA) was applied to the respective data. The analysis confirmed the unidimensional structure of the scale, consistent with the structure proposed by the authors who developed it. Sampling adequacy was good (KMO = 0.86), and Bartlett’s test of sphericity was statistically significant (*χ*^2^ (190) = 818.85, *p* < .001), indicating that the data were suitable for factor analysis. Internal consistency was excellent, with Cronbach’s alpha = 0.90 (Table 2). The Greek version of the Multifactorial Memory Questionnaire (MMQ) was used under license from the *Baycrest* Centre for Geriatric Care, Toronto.

#### 2.2.3. R4Alz-pc Index

The REMEDES for Alzheimer’s (R4Alz) battery is a novel neuropsychological tool designed to assess cognitive control, particularly in individuals who may be in preclinical stages of AD, such as those experiencing SCD or MCI. Developed by Poptsi et al. (2019, 2024), the R4Alz aims to detect early cognitive deficits associated with neurocognitive disorders. The battery assesses various cognitive domains, including working memory, attention, inhibitory control, and cognitive flexibility, through a series of tasks. Its development was guided by the need for ecologically valid and psychometrically sensitive tools with the potential to detect subtle cognitive deficits before the manifestation of clinical symptoms.

In the present study, only a subset of the full battery was employed, specifically the R4Alz-pc (preclinical) index. This decision was based on previous validation work by Poptsi et al. (2024), which demonstrated the index’s sensitivity in distinguishing between cognitively healthy older adults from those with SCD.

The R4Alz-pc index, a computerized version of the battery, operates through a digital interface incorporating visual, auditory, and motor modalities to create an interactive and adaptable assessment setting. Visual components consist of animal sketches (e.g., cat, dog, sheep) to which participants must respond (e.g., naming the animal once a light appears). Auditory input includes corresponding animal sounds, which serve either as confirmatory signals or as targets requiring specific responses (e.g., pressing the Enter key when hearing a dog bark). Light indicators (green or red) also serve as visual cues prompting participants to follow alternating color sequences while inhibiting responses to repeated colors. This multimodal system enables integrated testing of cognitive and sensorimotor performance in a standardized environment. Prior to each subtest, participants were given detailed instructions, often accompanied by a practice example when appropriate.

The tasks of the R4Alz-pc index are described below.

Inhibition task: This task evaluates how well someone can suppress irrelevant information or actions, and it consists of four conditions. In the first, participants name animal sketches, while in the second, they identify animal sounds. In the third and fourth conditions, participants must inhibit one stimulus modality: either name the animal while ignoring the sound or name the sound while ignoring the animal. Performance is scored from 0–60, where a higher score indicates more errors, thus worse performance.

Task/Rule-Switching task: This task assesses the ability to quickly adapt to changing demands or instructions, and it includes two conditions. In the first, participants switch from naming animal sounds to naming the image of an animal when a red light is activated. In the second condition, they must repeatedly switch between naming the animal sounds and the animal sketches by keeping a specific rule in mind, which is “when the white light is activated, name the sound you hear, and when the red light is activated, name the sketch you see”. The total score ranges from 0 to 63, with a higher score indicating worse performance.

Cognitive Flexibility task:

The first condition evaluates the integration of inhibitory control and task-switching, key aspects of cognitive flexibility. It comprises four sub-conditions in which participants are required to press the Enter key on the computer keyboard, based on concurrent visual (sketches) and auditory (animal sounds) stimuli. The procedure involves unexpected changes in the response rules, compelling participants to interpret performance-based feedback and adjusting their approach accordingly. For example, in the initial phase, participants must press the Enter key whenever a light appears on the image of a cat, while visual and auditory stimuli for different animals are simultaneously presented. In the subsequent phase, the rule changes, and participants are expected to respond solely to the cat’s sound. Scoring ranges from 0 to 56, where higher scores indicate worse performance. The second condition places additional demands on executive functions and includes four subtasks of increasing difficulty. In Condition B, participants are presented with a row of virtual lights displayed on the screen with alternating green and red colors. In step 1, participants asked to deactivate lights from left to right, following an alternating pattern (e.g., green, red, green), skipping repeated colors. In step 2, the pattern is reversed: lights are deactivated from right to left, beginning with red, again skipping any consecutive same-colored lights. Performance is evaluated based on the number of errors made during both steps. The score ranges from 0 (perfect performance) to 8 (maximum number of errors, with an error in each set).

The index was calculated by combining the individual component scores as described above. Each variable was normalized to fall within the [0,1] range using a min-max scaling method. The overall score of the index was computed by summing the squared values of the normalized variables, as shown below:

$$S_{Executive\;Functioning}={X_{ICT-RST\;1\&2}^{\prime }}^{2}+{X_{CFT}^{\prime }}^{2}+{X_{CFT\;cond\;b}^{\prime }}^{2}$$

This squaring process amplifies distinctions between high and low performance, giving more weight to superior outcomes (i.e., lower error scores) while reducing the impact of weaker ones. Each of the constituent variables contributes equally to the total index score, which ranges from 0 (indicating optimal performance) to 3, since min-max normalization scales all values between 0 and 1. This composite index demonstrated high diagnostic accuracy, achieving a sensitivity of 91.3% and a specificity of 82.4% in differentiating between cognitively healthy older adults and individuals with SCD, based on a cut-off score of 0.401 (Poptsi 2024).

The R4Alz-pc index has demonstrated robust psychometric properties. Previous validation studies have supported its construct validity, as well as its convergent validity through consistent associations with established neuropsychological tools assessing related cognitive domains. Additionally, it has shown high sensitivity and specificity in distinguishing between cognitively healthy aging and individuals in preclinical AD (Poptsi et al. 2020, 2023, 2024).

### 2.3. Procedure

Data collection was conducted in person over a three-month period, with 105 participants successfully completing the study protocol. At the outset of each session, and following the provision of informed consent, a unique personal code was generated for each participant by the research team to maintain anonymity while enabling accurate data linkage across measures. Participants completed demographic questionnaires, self-report measures, including the Positive and Negative Affect Schedule (PANAS) and the Multifactorial Memory Questionnaire (MMQ): Ability subscale, and the R4Alz-pc index, which served as the objective neuropsychological assessment. To mitigate potential order effects, the sequence of administration was counterbalanced across participants. All assessments were administered individually in a quiet environment, with the total session duration ranging between approximately 50 min and 1 h, and the R4Alz-pc index taking around 20 min to complete.

### 2.4. Ethics

The study was conducted in full compliance with the European Union’s General Data Protection Regulation (EU) 2016/679 (28 May 2018) and adhered to the principles outlined in the Declaration of Helsinki (World Medical Association 1997). All participants provided written informed consent after receiving detailed information about the study’s aims, their right to voluntary participation, and their ability to withdraw at any time. Participants did not receive monetary compensation for their participation. They were also informed that they could submit a written request to have their data removed from the online database. The research protocol was approved by the Scientific and Ethics Committee of the Greek Association of Alzheimer’s Disease and Related Disorders (Approval number: 96/20-12-2023).

### 2.5. Statistical Analysis

Statistical analyses were conducted using IBM SPSS Statistics (Version 29.0) and EQS 6.4 (Bentler 2020). Descriptive statistics were first calculated, including the mean (M) and standard deviation (SD) for the three primary variables: subjective memory estimations, trait affect, and performance on the R4Alz-pc index. Subsequently, Pearson correlation analyses were computed to examine the interrelations between subjective memory, affective traits, and objective cognitive control performance (R4Alz-pc index). Lastly, path analysis was performed to investigate the direct relationships specified in the proposed models.

Path analysis was conducted using Structural Equation Modeling (SEM) with maximum likelihood estimation. Model evaluation included the chi-square (χ^2^) test, where *p*-values above 0.05 indicate a good model fit. Additionally, the Root Mean Square Error of Approximation (RMSEA) was examined, with values below 0.05 indicating excellent fit and values between 0.06 and 0.08 considered acceptable, particularly for small samples (*n* < 100). The Comparative Fit Index (CFI) was used to assess model adequacy, with values above 0.90 reflecting acceptable fit and values close to 1.00 indicating excellent fit. Finally, Lagrange Multiplier and Wald tests were used to explore whether any model paths could be added or removed to improve overall model fit.

## 3. Results

Descriptive statistics for the main study variables are presented in Table 3. As noted, higher scores on the MMQ-Ability scale indicate better subjective memory estimates. Frequency analysis of objective assessment scores indicated that a substantial majority of the sample, 96 out of 105 participants (91% of the total sample), exhibited objective cognitive deficits, as defined by the test’s established cut-off score (see Instruments). Among them, approximately half of the participants (*n* = 54; 52.4%) showed only minimal impairment, with performance falling within the 0.5–1.4 range. Nine participants (9%) demonstrated performance within normal limits, suggesting intact cognitive control abilities. Notably, the nine participants with scores within normal limits represented a small subgroup; however, over half of the remaining sample scored only slightly below the cut-off, indicating that their performance was only marginally reduced. This suggests a relatively mild deviation from optimal cognitive control for the majority. Interestingly, these nine participants did not belong to the youngest age group and did not appear to differ from the rest of the sample in terms of basic demographic characteristics.

Pearson correlations indicate that positive trait affect was positively and statistically significantly associated with MMQ-Ability (*r* = 0.34, *p* < .001), indicating that individuals with higher levels of positive trait affect reported better subjective estimates about memory, whereas negative trait affect was negatively and statistically significantly associated with MMQ-Ability (*r* = −0.40, *p* < .001), suggesting that higher levels of negative trait affect were related to worse estimates. No significant relationships were found between positive or negative affect and objective cognitive control performance. Hence, this performance was not influenced by trait affect. Notably, the association between subjective memory estimations and objective cognitive performance was not statistically significant (see Table 4).

Given the N of the sample, separate path analyses regarding positive and negative affect were subsequently conducted to examine the direct relationships between trait affect, objective cognitive control performance, and subjective memory estimates.

**Figure 1:**
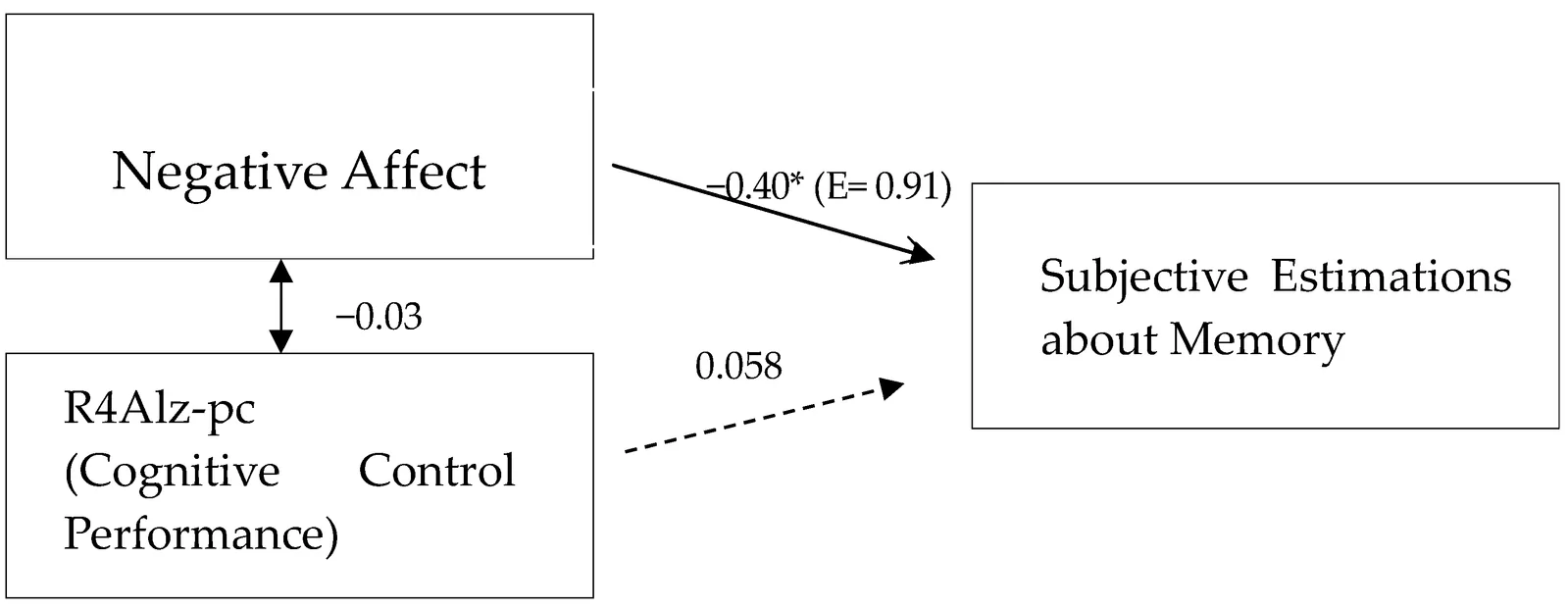
Path diagram illustrating the direct relationships of negative trait affect and objective cognitive control performance (R4Alz-pc index) with subjective memory estimations. * Standardized path coefficients and correlations are reported on the paths (*p* < 0.05). E = measurement error. Paths without an asterisk are non-significant.

The first path model, which was confirmed regarding negative affect, demonstrated excellent fit to the data, χ^2^(1) = 0.100, *p* > 0.05, CFI = 1.000, RMSEA = 0.000 (90% CI = 0.000–0.178). In this model, the independent variables were negative affect and R4Alz-pc, and the dependent variable was subjective estimations about memory. Results indicate that negative affect significantly predicted subjective memory estimations. Consistent with the bivariate correlations, higher levels of negative trait affect predicted worse memory estimations. Objective cognitive control performance did not significantly predict subjective memory estimations (see Figure 1).

**Figure 2:**
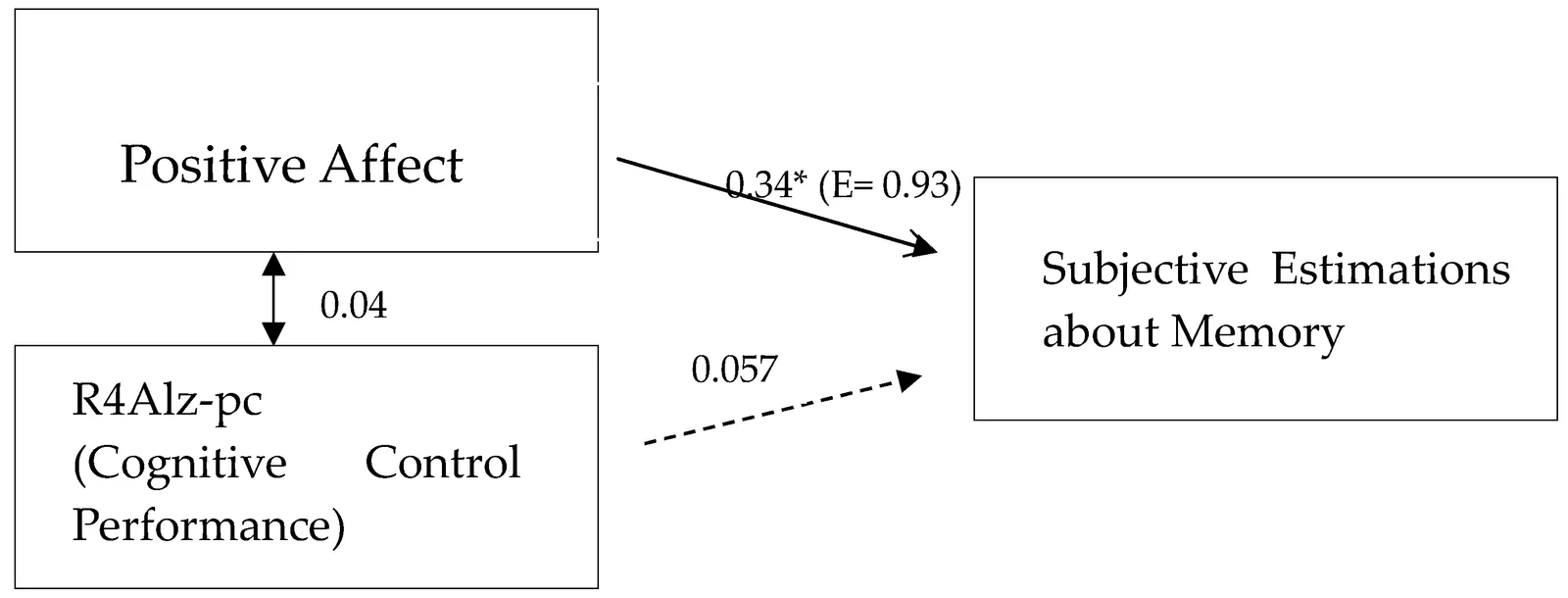
Path diagram illustrating the direct relationships of positive trait affect and objective cognitive control performance (R4Alz-pc index) with subjective memory estimations. * Standardized path coefficients and correlations are reported on the paths (*p* < 0.05). E = measurement error. Paths without an asterisk are non-significant.

The second path model which was confirmed regarding positive affect, has also exhibited excellent fit to the data, χ2(1) = 0.168, *p* > 0.05, CFI = 1.000, RMSEA = 0.000 (90% CI = 0.000–0.194). According to this model, the independent variables were positive affect and the R4Alz-pc index, and the dependent variable was subjective estimations about memory. The findings reveal that positive affect significantly predicted subjective memory estimations. Higher levels of positive affect were associated with better memory estimations. Consistent with the first model, objective cognitive control performance did not significantly predict subjective memory estimations (see Figure 2).

## 4. Discussion

The present study aimed to examine the relationship between subjective estimations of memory functioning, present in preclinical AD (SCD and MCI), and objective cognitive control performance in adults, with a specific focus on the role of trait affect as a potential explanatory factor. According to the existing theoretical framework, two hypotheses were tested: (1) trait affect would significantly predict subjective estimations of cognitive functioning, and (2) trait affect would significantly predict objective cognitive control performance.

Confirming Hypothesis 1, the findings show that trait affect emerged as a significant predictor of subjective memory evaluations. Participants with higher levels of negative trait affect tended to report worse estimations about their memory function, while participants with higher positive trait affect tended to have better estimations about their memory. Such findings are best understood within the broader framework of personality psychology, given that trait affect is conceptually linked to enduring personality dispositions (Watson et al. 1988). For instance, Sutin et al. (2020) examined the Five-Factor Model traits in relation to subjective cognitive failures. They reported that higher neuroticism is associated with more frequent subjective cognitive failures, whereas traits linked to positive affect and self-regulation, notably higher conscientiousness (and to some extent, agreeableness), correspond to fewer reported failures. These patterns imply that individuals prone to negative emotional states (neuroticism reflecting chronic worry and stress reactivity) tend to notice and complain more about cognitive lapses, whereas those who are more organized, self-disciplined, or emotionally stable report fewer lapses. Intriguingly, the same study found that controlling for depressed mood only partially attenuated these associations, suggesting that it is truly the enduring personality disposition, not just state affect, that influences subjective cognition. This observation resonates with present findings: even after accounting for current mood symptoms, trait affect as an aspect of personality likely continues to skew individuals’ self-appraisal of memory.

Further support comes from work on metacognitive accuracy. Colvin et al. (2018) examined how mood and personality characteristics relate to metamemory, the accuracy of one’s knowledge about their own memory performance (Troyer and Rich 2002). They identified two profiles among older adults: one profile with high positive traits (e.g., high extraversion and conscientiousness, low neuroticism/anxiety) and another with high negative affect traits (low extraversion/conscientiousness, high neuroticism/anxiety). These profiles differed in self-awareness of memory: the high-positive trait group demonstrated accurate self-appraisal of their memory abilities, whereas the high-negative affect group underestimated their memory performance despite performing objectively well. The authors concluded that these stable affective and personality patterns likely shape the schemas individuals use when evaluating themselves, creating biases in self-perception that may distort the accuracy of subjective estimations of cognitive functioning.

These findings carry significant clinical implications. When subjective cognitive complaints are used as a primary diagnostic criterion (e.g., SCD), an individual’s affective profile may systematically bias the process. Lenehan et al. (2012) found that the inclusion of subjective complaints in MCI diagnostic models decreased diagnostic accuracy, increasing rates of both false-positive and false-negative diagnoses. High negative trait affect may cause overreporting, leading clinicians to mistakenly identify impairment where none exists, while a highly positive affective profile may result in underreporting, delaying the recognition of early decline. Thus, reliance solely on subjective reports risks misdiagnosis: in some cases, triggering unwarranted interventions, and in others, missing critical opportunities for early treatment.

On the other hand, Hypothesis 2 was not confirmed since trait affect did not significantly predict objective cognitive control performance, suggesting that it primarily influences subjective cognitive estimations rather than actual cognitive control abilities. This finding, in addition to the aforementioned results, contributes to a growing body of evidence highlighting the dissociation between subjective and objective cognitive measures (Crumley et al. 2014; Minett et al. 2008; Zlatar et al. 2018), and the role of personality-related factors in this divergence (Colvin et al. 2018; Costa et al. 2023). Recently, Costa et al. (2023) demonstrated that certain personality traits can moderate the link between subjective and objective cognition in older adults. In their study, high extraversion and low agreeableness were associated with the greatest subjective–objective discrepancies: notably, individuals who were more extraverted and less agreeable had worse objective cognitive performance, yet reported the fewest memory complaints. This mirrors our observation that a highly positive affective style can mask cognitive deficits when they are estimated subjectively. Furthermore, individuals with low levels of agreeableness may tend to present themselves in a less modest way when reporting their memory abilities (Hülür et al. 2015). Furthermore, Williams et al. (2010) suggest that these individuals might also have reduced cognitive capacity to regulate their behavior according to social norms, due to difficulties with impulse control. This impulsivity has been linked to lower performance on objective cognitive tasks (Williams et al. 2010). As a result, the combination of inflated self-perception (due to low modesty) and actual cognitive deficits could lead these individuals to overestimate their cognitive abilities (Costa et al. 2023).

Unlike self-reported questionnaires, a performance-based test like the R4Alz-pc AD index directly measures cognitive abilities through standardized tasks (Poptsi et al. 2024). This means that it directly measures the accuracy of a response to a stimulus, without involving the individual’s subjective estimation. Performance depends on neurocognitive processes and not on the person’s psychological state or personality. The independence of this measurement from the affective profile is critical for its potential use as a highly reliable screening tool for the early detection of cognitive deficits. In cases where subjective estimations may be masked or exaggerated by an individual’s emotional disposition, an objective measure provides a clearer and unbiased picture of cognitive status. This is crucial for early intervention and treatment, as executive function deficits are well-established as some of the earliest cognitive markers of the preclinical or prodromal stages of neurodegenerative diseases (Huff et al. 2015; Pantsiou et al. 2018; Poptsi et al. 2024; Tsentidou et al. 2022).

While our study did not find a direct predictive relationship between trait affect and objective cognitive control performance, it is crucial to consider the broader theoretical and empirical literature that suggests a dynamic and complex interplay between affect and executive functions. The well-documented “positivity effect”, a tendency among older adults to prioritize and better recall emotionally positive over negative stimuli, is often considered a motivational shift driven by emotion-regulatory goals (Carstensen and Mikels 2005). These shifts, in turn, could influence cognitive control processes. For instance, research has shown that older adults often allocate attentional resources preferentially to positive stimuli, a selective process that is mediated by the frontoparietal cognitive control network (Mather and Carstensen 2005). In line with this, affective regulation strategy may reflect or even reinforce changes in personality traits with age, which, in turn, could dynamically impact the efficiency of cognitive control.

The fact that we found these prevalent, objectively measurable deficits that are not predicted by trait affect provides a robust argument for prioritizing objective assessment. Therefore, the R4Alz-pc index appears to offer a sensitive means of identifying preclinical cognitive decline from its very beginning through objective metrics. Clinically, this index could serve as a valuable screening instrument for individuals of advancing age in the general population, facilitating the early recognition of cognitive changes at a stage when they may still be modifiable through targeted interventions or lifestyle adjustments.

Overall, these findings highlight the importance of considering trait affect when interpreting subjective estimates about memory in older adults. While subjective reports offer useful insights, they may be biased by affective dispositions. To our knowledge, this is the first study to examine how trait affect relates to both subjective and objective cognition, underscoring the value of objective assessments as a more stable and reliable indicator of cognitive status even from the beginning of decline.

### 4.1. Limitations of the Study

This study has some limitations that should be considered. First, the relatively small sample size may limit the generalizability of the findings. It should also be noted that the current sample was highly educated, which should be considered when interpreting the results. Additionally, only specific personality characteristics were examined, while other potentially relevant traits were not assessed. Moreover, the relationship between trait affect and cognition was explored using selected subjective and objective measures; different tools might yield complementary results. Future studies should address these limitations to better understand the relationship between stable affective characteristics, subjective estimations, and objective performance.

### 4.2. Future Research Recommendations

Future studies should employ longitudinal designs to more clearly explore the causal relationships between trait affect and subjective cognitive estimations over time. Expanding sample sizes and including more diverse populations would improve generalizability. Finally, incorporating clinical, neuroimaging, and biomarker data could help disentangle whether early subjective complaints reflect true neurodegenerative processes, cardiovascular issues or trait-related perceptual biases.

## 5. Conclusions

The present study highlights the critical role of objective cognitive assessment of cognitive decline in its beginning in middle-aged and young-old adults, especially in light of the distortive influence that trait affect may exert on subjective estimations usually used to detect SCD. While self-reported cognitive complaints are valuable, they can be biased by stable emotional dispositions. The R4Alz-pc index proved to be a sensitive and reliable tool, detecting subtle executive dysfunction even in participants who were considered healthy and cognitively intact. Its use may enhance early detection of cognitive decline due to cardiovascular factors and neurodegeneration, supporting timely intervention efforts. Integrating objective tools like R4Alz-pc into research settings can also provide a more accurate assessment of cognitive health in aging.

## Figures and Tables

**Table 1:** Demographic characteristics of the sample.

Variable	*n*	*%*	M	SD	Range
Gender					
Female	70	66.7			
Male	35	33.3			
Age (years)			59.25	5.21	45–74

**Table 2:** Factor loadings of the MMQ-Ability scale.

	1
18. You misplace something that you put away a few days ago.	0.774
13. You forget to pass on a message.	0.696
5. You leave something behind when you meant to bring it with you.	0.695
20. You forget details about a recent conversation.	0.663
8. You forget to run an errand.	0.659
19. You forget to buy something you intended to buy.	0.656
15. You forget a birthday or anniversary that you used to know well.	0.637
7. You forget what you were just about to do; for example, walk into a room and forget what you went there to do.	0.632
2. You misplace something you use daily, like your keys or glasses	0.626
14. You forget what you were going to say in conversation	0.609
6. You forget an appointment.	0.605
10. You have trouble remembering details from a newspaper or magazine article you read earlier that day.	0.592
1. You forget to pay a bill on time.	0.550
9. You have difficulty coming up with a specific word that you want.	0.547
17. You retell a story or joke to the same person because you forgot that you had already told him or her.	0.545
16. You forget a telephone number you use frequently.	0.513
3. You have trouble remembering a telephone number you just looked up.	0.479
11. You forget to take your medication.	0.478
12. You cannot recall the name of someone you have known for some time.	0.447
4. You cannot recall the name of someone you just met.	0.433

**Table 3:** Mean, standard deviation and range for study variables.

Variable	Μ	SD	Range
MMQ-Ability	54.11	10.47	22–79
Negative Trait Affect	21.97	6.67	10–39
Positive Trait Affect	34.79	7.04	19–49
R4Alz-pc	1.30	0.82	0.07–4.73

**Table 4:** Pearson correlations among study variables.

	1	2	3	4
1. MMQ-Ability	1			
2. Negative Trait Affect	−0.40 **	1		
3. Positive Trait Affect	0.34 **	−0.35 **	1	
4. R4Alz-pcAD	0.07	−0.03	0.04	1

Note: ** = *p* < 0.01. Correlations without asterisks are not statistically significant.

## Data Availability

The original contributions presented in this study are included in the article. Further inquiries can be directed to the corresponding author.
